# Invitation for Nominations and Applications for the 2022 Scientific Achievement Awards, the ASN Class of 2022 Fellows, and Other Honors

**DOI:** 10.1093/cdn/nzab143

**Published:** 2021-11-23

The American Society for Nutrition Foundation (ASNF) awards program honors scientists, clinicians, and scholars for significant achievements in nutrition research and practice. Nominations/applications are now being accepted for the 2022 Scientific Achievement Awards, ASN Class of 2022 Fellows, and other honors.

## Nomination/Application Instructions

- Enter the ASN Foundation Portal (https://nutrition.org/asn-foundation/awards/) to submit your nominations and applications.
- The deadline for receipt of nominations and applications is **28 January 2022**.
- Each nomination for a scientific achievement award or application for the ASN Class of 2022 Fellows must include *1*) a letter of support outlining the basis for the nomination and the significance of the candidate's work, and *2*) a curriculum vitae for the nominee/applicant, including a list of the nominee's or applicant's publications in refereed journals. Please note that additional requirements for ASN Class of 2022 Fellows can be found at https://nutrition.org/asn-foundation/fellows/.
- All nomination or application materials must be submitted electronically through the ASN Foundation Portal. Incomplete or late submissions will not be considered. For the most up-to-date information regarding the Society awards program, visit https://nutrition.org/asn-foundation/awards/. ASN membership is not required to submit a nomination.
- Nominations for scientific awards and applications for the ASN Class of 2022 Fellows will be retained for 2 y.
- Applications for fellowships, scholarships, and other funding opportunities will not be retained for future years and should be submitted on an annual basis.
- An individual who has received 1 ASN award is not eligible to receive another award unless it is for accomplishments not previously recognized.
- ASN membership is not a requirement for all Society awards but outlining service to the Society is helpful when applicable.
- Any questions, please contact awards@nutrition.org.

## Selection Process

- Confidential award selection juries are appointed each year. Juries rank the candidates based on the award criteria.
- An award is usually given to 1 person, but a jury may recommend that the award be given to ≥2 individuals who collaborated in recent research. Similarly, a jury can determine that there are no deserving candidates in a given year.

## Young Investigator Awards

### The Bio-Serv Award in Experimental Animal Nutrition

This award is given for meritorious research in nutrition accomplished by an investigator within 10 y of postgraduate training. The work recognized must involve the nutrition of experimental animals used as models. The award is traditionally supported by Bio-Serv, Inc.

Recent recipients include A Steelman, V Singh, Z Madak-Erdogan, K Habegger, RS Rector.

### The ELR Stokstad Award

This award is given for outstanding fundamental research in nutrition, with preference to scientists at relatively early stages in their careers. The award is supported by an endowment from the ELR Stokstad family.

Recent recipients include A Das, K Shea, Y Ah-Seo, M Vijay-Kumar, JM Brown.

### The Mary Swartz Rose Young Investigator Award

This award is given to an investigator within 10 y of postgraduate training, for outstanding research on the safety and efficacy of bioactive compounds for human health. The award is traditionally supported by the Council for Responsible Nutrition.

Recent recipients include N Moran, J Amengual, A Das, S Raza Shaikh, S Wang, GD Noratto.

### The Mead Johnson Award

This award is given to an investigator for a single outstanding piece of nutrition research or a series of papers on the same subject accomplished within 10 y of postgraduate training. The award is endowed by the Mead Johnson Pediatric Nutrition Institute.

Recent recipients include H Holscher, AW Brown, H Eicher-Miller, R Seguin, KA Varady.

### The Norman Kretchmer Memorial Award in Nutrition and Development

This award is given to a young investigator for a substantial body of independent research in the field of nutrition and development with potential relevance to improving child health. ASN seeks funding partners to support this award.

Recent recipients include T Swindle, S Mehta, AL Thompson, C Stewart, K Claycombe.

### The Peter J Reeds Young Investigator Award

This award is given for outstanding research in macronutrient metabolism accomplished within 5 y of receiving a Ph.D. degree or completing residency training. In the case of M.D. candidates, eligibility includes time of completing residency training or postdoctoral fellowship. This award was established with an initial contribution from the Children's Nutrition Research Center at Baylor College of Medicine. Nominees must be ASN members. ASN seeks funding partners to support this award.

Recent recipients include L Bowers, TA Churchward-Venne, H Dashti, T Voortman.

### The Samuel J Fomon Young Physician Award

This award is given to a physician within 10 y from completion of medical postdoctoral training for outstanding work in the general area of pediatric nutrition, including infant nutrition, infant growth, or body composition. Because the award honors Dr. Fomon, preclinical and/or clinical research that contributes to medical applications or produces impacts upon the practice of infant feeding will be recognized. The award is endowed by the Nestlé Nutrition Institute.

Recent recipients include D Young Lee, A Salas, AB Hair, DE Roth.

### The Vernon Young International Award for Amino Acid Research

This award is given to an investigator within 10 y of postgraduate training for a single outstanding piece of research or for a series of papers in a related area of amino acid metabolism. The award is endowed by the Ajinomoto Health & Nutrition North America, Inc.

Recent recipients include M Dirks, D Trico, BS Gordon, E Bejarano, SM Pasiakos, MJ Drummond.

## Mid-Career Awards

### The Nevin Scrimshaw Mid-Career Award in Global Nutrition

This award is given to a mid-career professional who has done innovative work to advance the field of global nutrition and is a current member of ASN's Global Nutrition Council. The awardee will be between 10 and 20 y post-terminal degree and have a sustained record of substantial research, mentoring, and training. There are no geographic restrictions, and awardees may come from academia, public service, or the NGO or private sector. The award is supported by a grant from the Sight and Life Foundation and contributions from Global Nutrition Council members.

Recent recipients include A Gernand, P Menon, LE Murray-Kolb, L Neufeld.

## Senior Investigator—Educator & Mentor Awards

### ASN Foundation Mentorship Award

This award is given for outstanding mentorship in the development of successful nutrition science investigators. Nominations should include a description of the nominee's mentoring skills and evidence of mentorship (joint publications grant funding with trainee, etc.), a list of current and former trainees and the positions they have achieved, and additional support letters from trainees (a minimum of 2 and a maximum of 5). ASN seeks funding partners to support this award.

Recent recipients include B Rolls, JM Ordovas, P Kris-Etherton, B Shane, EH Jeffery.

### The Excellence in Nutrition Education Award

This award is given for outstanding contributions to teaching nutrition. The recipient will have demonstrated current superior ability as an educator in nutrition science as evidenced through a combination of the following: acknowledged excellence in nutrition teaching or nutrition education research and original contributions to teaching and translating new discoveries into classroom or educational materials. The award is traditionally supported by the Nestlé Nutrition Institute.

Recent recipients include M Kohlmeier, S Hursting, M McGuire, EJ Mayer-Davis, C Byrd-Bredbenner.

### The Roland L Weinsier Award for Excellence in Medical/Dental Nutrition Education

This award is given in recognition of an outstanding career in medical/dental nutrition education. The nominee's efforts should be widely recognized and have had a national or international impact. Nominations should acknowledge excellence in nutrition teaching or nutrition education research that extends beyond the local institution and includes innovations in medical/dental education. ASN seeks funding partners to support this award.

Recent recipients include TA Marshall, C Lenders, CA Palmer, D Seidner.

## Senior Investigator Awards

### ASN Nutritional Sciences Award

This award is given for recent investigative contributions of contemporary significance to the understanding of human nutrition. The contributions need not be restricted to investigative work with humans as long as they have relevance to human nutrition and health. Preference is usually given to scientists in the Western hemisphere. ASN seeks funding partners to support this award.

Recent recipients include D Allison, KL Tucker, W Campbell, MR Forman, S Booth.

### Conrad A Elvehjem Award for Public Service in Nutrition

This award is given for specific and distinguished service to the public through the science of nutrition. Such service could be rendered through governmental, industrial, private, or international institutions, but contributions of an investigative character are not excluded. The award is not limited to ASN members. Nominations should include a selected bibliography indicating the candidate's contributions to public service. The award is traditionally supported by Mondelēz International.

Recent recipients include B Schneeman, AH Lichtenstein, J Erdman, KM Rasmussen, NC Nebeling.

### The David Kritchevsky Career Achievement Award

This award is presented in recognition of a career devoted to promoting interaction among, support for, and assistance of outstanding nutrition researchers in governmental, private, and academic sectors resulting in the application of fundamental knowledge to delivery of better nutrition products and information to the public. The award is traditionally supported by Mondelēz International.

Recent recipients include D Klurfeld, AF Subar, Jose Ordovas, CM Weaver, AH Lichtenstein.

### The EV McCollum Award

This award is given to a clinical investigator who is perceived as a major creative force, actively generating new concepts in nutrition and personally seeing to the execution of studies testing the validity of these concepts. ASN seeks funding partners to support this award.

Recent recipients include CS Mantzoros, NE Deutz, S Booth, KD Hall, J Mason.

### General Mills Bell Institute of Health and Nutrition Innovation Award

This award recognizes an investigator whose scientific contributions advance the understanding of healthy dietary patterns, which include whole grains, cereals, fruits, vegetables, and/or dairy. Eligible recipients are conducting mechanistic, epidemiological, clinical, and/or translational research contributing to the understanding of the benefits of a healthy dietary pattern. The award is endowed by the General Mills Bell Institute of Health & Nutrition.

Recent recipients include J Reedy, S Roberts, M Ferruzzi, S Liu, RH Liu.

### The Mary Swartz Rose Senior Investigator Award

This award is given to an investigator for outstanding research on the safety and efficacy of bioactive compounds for human health. The award is traditionally supported by the Council for Responsible Nutrition.

Recent recipients include C Brenner, C Ross, E Johnson, GM Pasinetti, JB Blumberg.

### The McCormick Science Institute Research Award

This award is presented to an investigator conducting clinical, translational, in vitro, and/or ex vivo research, whose scientific contributions have advanced the understanding of the potential health benefits of culinary herbs and spices. The award is endowed by the McCormick Science Institute.

Recent recipients include Z Li, RA Anderson, JC Peters, KH Kim, J-M Zing.

### The Osborne and Mendel Award

This award is given for outstanding recent basic research accomplishments in nutrition. The award is traditionally supported by ILSI North America.

Recent recipients include J Speakman, J Zempleni, V Gladyshev, JT Brenna, RS Eisenstein.

### Robert H Herman Memorial Award

This award is given to a clinical investigator whose research work has contributed importantly to the advancement of clinical nutrition, particularly the biochemical and metabolic aspects of human nutrition. ASN seeks funding partners to support this award.

Recent recipients include T Wolever, J Selhub, C Mantzoros, DJA Jenkins, S Klein.

## Senior Investigator Lectureships

Recipients of the following 4 awards will deliver a lecture on a topic consistent with the award's purpose at NUTRITION 2022 LIVE ONLINE, ASN's flagship meeting which will be held virtually on 14–16 June, 2022.

### The EV McCollum International Lectureship in Nutrition

This biannual award provides a means to encourage sound advancements in nutritional science and their application for improving the health and well-being of people worldwide and to commemorate the life and contributions of EV McCollum. The lectureship is endowed by the EV McCollum International Lectureship Endowment Fund.

Recent recipients include E Frongillo, A Stein, RJ Stoltzfus, KG Dewey, L Allen.

### George Bray Outstanding Scientific Achievements Award in Obesity Research

This award will be presented to a physician, clinician, or investigator who has made a significant lifetime contribution to the field of obesity research. This award is designed to recognize an individual whose lifetime body of work includes meaningful contributions and/or demonstration of highly original, sustained, and reproducible scholarship that has made major contributions to the understanding of the causes, treatment, and/or prevention of obesity. The award is endowed by Dr. George A Bray and Marilyn M Bray.

Recent recipients include E Ravussin.

### Lifetime Achievements in Global Nutrition Research Award

This award honors lifetime achievements in international nutrition research to benefit populations in nonindustrialized countries. Nominations should describe the nominee's contributions to research, as demonstrated through peer-reviewed publications, and training new scientists for international nutrition research. Nominees must be a member of the Global Nutrition Council. ASN seeks funding partners to support this award. (Formerly called the Kellogg Award for Lifetime Achievements in International Nutrition Research.)

Recent recipients include R Perez-Escamilla, MT Ruel, C Geissler, BL Rogers, ME Bentley.

### The Robert Suskind and Leslie Lewinter-Suskind Pediatric Nutrition Lifetime Achievement Award

This award is presented to a clinician or investigator who has made a significant lifetime contribution to the field of pediatric nutrition, particularly to the role of childhood nutrition in health and disease. The recipient's impact will have been global, resulting in scientifically verified improvements in children's health and/or innovative successful treatments for conditions such as childhood malnutrition or obesity. The award is endowed by the Suskind family.

Recent recipients include V Stallings, B Lönnerdal, RE Black, KH Brown, N Krebs.

## ASN Fellows

The ASN Fellows Selection Panel invites applications for the ASN Class of 2022 Fellows. The ASN Fellow designation is the highest honor bestowed by the Society in recognition of significant discoveries and distinguished careers in nutrition. Scientists who have had distinguished careers in the field of nutrition and are ≥65 y of age are eligible to apply for consideration.

For more information on the ASN Fellows program, please visit https://nutrition.org/asn-foundation/fellows/.

## Other Awards

### ASN Industry Recognition Award

This award recognizes significant contributions to the field of nutrition science or practice by an individual from the industry sector (includes commodity boards and industry trade organizations). This award is supported by ASN's Sustaining Partner Roundtable.

Recent recipients include A Wong.

### Korean Nutrition Society (KNS) Award

Established by the Korean Nutrition Society (KNS) and ASN in 2010 to improve understanding and cooperation between the societies in nutritional matters of common interest and concern, the KNS Award is presented each year to an ASN member scientist. The KNS Award promotes excellence in nutrition research conducted by a North American scientist who is an ASN member and engaged with KNS, Korea, or related collaborators or studies. Nominees for this award can include, but are not limited to, ASN members collaborating with KNS investigators; scientists studying issues relevant to the Korean population; Korean investigators involved in research in North America; and/or other ASN members working on outstanding research in nutrition.

Recent recipients include C Mantzoros, N Moustaid-Moussa, S Chung, H Han, NV Dhurandhar.

### ASN Volunteer of the Year Award

This award recognizes outstanding contributions to enhance, promote, and support the activities and programs of ASN.

Recent recipients include Members of ASN's Minority and Diversity Affairs Committee, D Bier, S Murphy, S Booth, DM Klurfeld.

## Scholarships, Fellowships, and Other Funding Opportunities

### Ajinomoto Young Investigator Pilot Grant

This award provides a one-time $10,000 pilot grant for a young investigator to pursue human research that advances the understanding of dietary glutamic acid and/or its sodium salt, under healthy or therapeutic conditions, particularly as it relates to sodium reduction and/or the gut–brain axis. No indirect costs are allowed. This funding opportunity is supported by Ajinomoto Health & Nutrition North America, Inc.

Recent recipients include S Galyean.

### ASN Foundation Predoctoral Fellowships

The ASN Foundation Predoctoral Fellowships fund outstanding research projects proposed and conducted by ASN members enrolled in a US graduate program in nutrition. Fellowship grants range from $2000 to $5000. This fellowship is endowed by The Gerber Foundation.

Recent recipients include C Klobodu, A Shrestha, K Harper, B Keirns.

### Herbalife Nutrition Scholarship

The ASN Foundation will award a $5000 scholarship to support the research and/or clinical training of a promising student seeking an advanced degree in nutrition or a related field. The scholarship is directed to Registered Dietitians (RDs) or those intending to become an RD. This scholarship is supported by Herbalife Nutrition.

Recent recipients include M Alan Dawson, K Ellison, N Litwin.

**Special Awards & Competitions**

ASN offers additional awards for students, faculty, clinicians, and researchers. We welcome your applications and sponsorship inquiries. Please visit the ASN website for more information, application deadlines, and sponsorship opportunities.

- WO Atwater Lectureship (contact: Stephanie Pearl, stephanie.pearl@usda.gov, USDA Agricultural Research Center)
- Milton L Sunde Award (for publication in *The Journal of Nutrition* of outstanding experimental, applied, or fundamental research in nutrition that uses an avian species, visit: https://academic.oup.com/jn)
- For students, postdoctoral fellows, and early career investigators:
  Clinical Emerging Leader Award Competition
  Early Career Research Award Competition (pending funding)
  Emerging Leaders in Nutrition Science Abstract Recognition Program
  Global Nutrition Early Career Scholar Award
  Graduate Student Research Award Competition
  Nutrition Translation Award Competition for Early Career Investigators
  Postdoctoral Research Award Competition, endowed by IFF
  Science Policy Fellowships
  Student Interest Group (SIG) Student Award
  Young Minority Investigator Oral Competition

## Notes

Address correspondence to MC (e-mail: awards@nutrition.org).

